# Editorial: Immuno-metabolic interactions and cancer progression in the tumor microenvironment

**DOI:** 10.3389/fimmu.2026.1774012

**Published:** 2026-01-13

**Authors:** Vincenzo Flati, Jacopo Di Gregorio, Nahum Puebla-Osorio, Khosrow Kashfi

**Affiliations:** 1 Department of Biotechnological and Applied Clinical Sciences, University of L’Aquila, L’Aquila, Italy; 2 Department of Radiation Oncology and The Institute for Data Science in Oncology, The University of Texas MD Anderson Cancer Center, Houston, TX, United States; 3 Department of Molecular, Cellular & Biomedical Sciences, City University of New York School of Medicine, New York, NY, United States; 4 Graduate Program in Biology, City University of New York Graduate Center, New York, NY, United States

**Keywords:** cancer, cancer immunity & immunotherapy, cancer metabolism, immuno-metabolic crosstalk, tumor microenvironment - TME

## 1 Introduction

Cancer metabolism underwent a conceptual revolution over the past decade. Research has long focused on aerobic glycolysis, but recently a more nuanced view is emerging. Tumors dynamically switch among different metabolic pathways, from oxidative phosphorylation to glutaminolysis and β-oxidation. Those metabolic alterations lead to increased growth and therapy resistance. Nevertheless, these alterations are not solely cell-autonomous. The tumor microenvironment (TME) acts as an active metabolic ecosystem, in which immune, stromal, and endothelial cells influence and respond to tumor metabolic demands.

The cross-talk between immunity and metabolism, referred to as *immunometabolism*, determines how tumors evade immune surveillance or how immune activation reshapes cancer metabolism. This Research Topic in *Frontiers in Immunology* brings together five papers (three reviews and two original research articles) exploring these interconnections across diverse malignancies, from squamous and lymphoid cancers to lung and laryngeal carcinomas. These studies provide insight into how metabolism cues within the TME-sculpt immune function and influence cancer fate.

## 2 Thematic overview of the contributions

### 2.1 Tumor-specific immunometabolic landscapes

In their review, Dong et al. analyze *cutaneous squamous cell carcinoma (cSCC)* as a paradigm of immune dysregulation within a metabolically altered TME. They highlight how UV-induced mutagenesis and chronic inflammation generate a immunosuppressive niche dominated by regulatory T cells, myeloid-derived suppressor cells, and tumor-associated macrophages. Mechanistic insight into the PD-L1/PD-1 and TGF-β/Smad pathways reveals how these axes integrate metabolic stress with immune escape. The review also summarizes clinical data on immune checkpoint inhibitors, including the monoclonal antibodies cemiplimab and pembrolizumab. The authors propose future strategies, including combinatorial and vaccine-based immunotherapy, tailored to the metabolic and immune profile of cSCC.

### 2.2 Integrating metabolism and immunity in lymphoma

Chen et al. provide a review on the *metabolic–immune axis in diffuse large B-cell lymphoma (DLBCL) and follicular lymphoma (FL)*. They emphasize that enhanced glycolysis and amino acid metabolism fuel tumor growth, but concurrently shape an immunosuppressive milieu *via* lactate accumulation, nutrient competition, and checkpoint evasion, with a molecular network involving PD-L1, LAG-3, and TIM-3. Their synthesis of PET-based metabolic imaging and immune profiling data demonstrates that integrating metabolic and immune biomarkers refines prognostic stratification and supports the rational combination of metabolic inhibitors with immunotherapy. This represents an important step toward precision lymphoma management.

### 2.3 Reimagining therapeutics through drug repurposing

Dong et al. propose a review focusing on *drug functional remapping* as a strategic approach to accelerate the development of cancer immunotherapy. By reanalyzing clinically approved drugs, the authors reveal how these agents may reprogram immune metabolism or counteract checkpoint inhibitor resistance. This review also surveys computational and high-throughput screening tools enabling systematic repurposing. The result is a compelling case for expanding therapeutic discovery beyond *de novo* drug development, using immunometabolic principles to reposition well-characterized compounds for cancer treatment.

### 2.4 Novel immunometabolic mechanisms uncovered

Li et al. identify *fibrinogen alpha chain (FGA)* as a suppressor of *lung adenocarcinoma (LUAD)* progression by modulating of *SLC7A11/xCT-mediated disulfidptosis*. Using transcriptomic analyses and functional assays, they demonstrate that FGA restrains malignant proliferation and invasion while influencing immune infiltration patterns in the LUAD TME. The study highlights disulfidptosis (a recently described disulfide-stress–induced cell death pathway) as a therapeutic vulnerability that links redox homeostasis to tumor immunity.

### 2.5 Metabolic subtyping as a clinical tool

Zheng et al. have combined integrative multi-omics and machine learning to delineate two metabolic subtypes of laryngeal cancer (LCA), with distinct prognoses and immune features. The LCA1 subtype exhibits enhanced metabolic pathway enrichment and NSD1 mutations, both of which are associated with improved outcomes. On the other hand, the LCA2 subtype shows higher immune infiltration and checkpoint expression but poorer survival. The diagnostic and prognostic models developed by the authors demonstrate strong predictive performance, reinforcing the clinical potential of metabolic profiling for patient stratification.

## 3 Emerging insights and unifying principles

Across these studies, several unifying themes that reshape our understanding of cancer immunometabolism can be identified. First, the reciprocal regulation between tumor and immune cells, which are metabolically interdependent. Tumor-derived metabolites (lactate, kynurenine, adenosine) modulate immune effector function, while activated immune cells impose metabolic pressure on tumor survival pathways.

Moreover, distinct metabolic states coexist within the same tumor, creating spatially and temporally heterogeneous niches that complicate therapy. Integrative -omics and spatial transcriptomic tools are beginning to unravel this complexity, but this will represent an important field of future research.

From a therapeutic standpoint, metabolic reprogramming can be leveraged to potentiate immunotherapy. From repurposed metabolic drugs to redox-targeted interventions and metabolic subtype-based treatment design, these strategies promise to overcome resistance and enhance immune efficacy. Analyzing metabolic, immune, and clinical datasets enables predictive modeling of patient outcomes, advancing immunometabolism toward actionable clinical translation.

## 4 Future directions

The next frontier in this field lies in dissecting the spatial and temporal organization of metabolic networks within the TME. High-resolution single-cell and spatial metabolomic technologies can now map nutrient fluxes and redox gradients at the cellular level, offering unprecedented insight into local metabolic competition and cooperation. Parallel efforts should focus on linking systemic metabolism and its pathological alterations, including obesity, diabetes, and cachexia, to intratumoral immunometabolic states; this would provide a more holistic understanding of host–tumor interactions.

Therapeutically, the challenge is to move from descriptive metabolic profiling to mechanistic intervention. Rationally designed combinations of metabolic inhibitors, checkpoint blockade, and drug-repurposing candidates could exploit specific vulnerabilities within the immunometabolic network. The integration of multi-omics data and AI-driven drug discovery platforms will further accelerate this transition from bench to bedside.

## 5 Concluding remarks

This Research Topic brings together diverse but complementary perspectives on how metabolism and immunity co-evolve within the tumor microenvironment. These contributions reinforce a central principle: the immune response to cancer cannot be understood apart from metabolism, and tumor metabolism cannot be targeted without considering its immune context.

By continuing to explore this convergence, we move closer to realizing the promise of a defined immunometabolic therapy. Targeting the metabolic circuitry of the TME enhances cancer control and restores immune balance.

